# Factors Influencing Mobility Relative to Nutritional Status among Elderly Women with Diabetes Mellitus

**Published:** 2018-06

**Authors:** Eun Ju LIM

**Affiliations:** Red Cross College of Nursing, Chung-Ang University, 84 Heukseok-ro, Dongjak-gu, Seoul, Republic of Korea

**Keywords:** Aged, Diabetes mellitus, Mobility, Nutritional status

## Abstract

**Background:**

The prevalence of functional disability is very high among elderly people. Malnourished individuals are less likely to recover from limited mobility and have higher risk of deterioration of functional disability. This study investigated factors influencing mobility relative to nutritional status in elderly women with diabetes mellitus.

**Methods:**

We analyzed data of 464 elderly women with diabetes mellitus, obtained from the 2014 cross-sectional survey of the Korea Institute for Health and Social Affairs.

**Results:**

In the well-nourished group, body mass index, difficulties in daily activities due to visual acuity loss, muscle strength, and depressive symptoms were significant predictors of mobility; the explanatory power of this model was 24.0% (*F*=12.905; *P*<0.001). In the malnourished group, muscle strength, depressive symptoms, and nutritional status were significant predictors of mobility; the explanatory power of this model was 40.5% (*F*=16.589; *P*<0.001).

**Conclusion:**

The level of mobility relative to nutritional status varied widely among elderly women with diabetes mellitus. Therefore, to improve mobility in malnourished elderly people, public health nurses must provide comprehensive nutrition management and a support system in addition to physical and psychological interventions.

## Introduction

Elderly people are at a high risk of nutrient deficiency from decreased food intake, a consequence of reductions in activity, digestive functions, and taste sensations (1). The overall nutritional status of elderly women is worse than that of elderly men (2), and nutritional conditions deteriorate with age (3). Malnourishment expedites the onset and courses of functional disabilities and mobility limitations (4) and leads to muscle and weight loss, as well as deteriorating health. Thus, malnourished patients eventually have difficulty recovering from chronic diseases (5). Malnourishment can increase dependency and demands for care among elderly people, leading to weakness and reduced mobility (6), as well as a decreased quality of life (7).

Nutritional status plays an important role in the occurrence, progression, and treatment of diabetes mellitus (DM) (1, 8). Antioxidants are an important component of the physical defense system. Antioxidant deficiency causes oxidative stress, which increases the incidence of DM and related complications (9). With increasing age, general decline in lower extremity strength can reduce mobility and the ability to perform daily activities, such as walking, standing up from a chair, and climbing stairs (10). In the elderly, nutritional problems can affect mobility and are associated with psychological factors such as depression and loneliness (11). Patients with DM may also experience the added burden of negative emotions, including fear, anxiety, depression, and feelings of helplessness regarding diabetic complications, combined with the pressure of blood glucose control (12). Such emotions make it difficult for patients with DM to maintain blood glucose control, leading to deteriorating health (12). In addition, patients may experience discomfort from diabetic complications in their daily lives, as well as aging-related vision loss (13). Elderly individuals who experience a decreased ability to live independently consequent to losses of function and mobility may experience complications caused by fracture, surgery, and immobility (14).

Nutritional evaluation is particularly important for elderly women, who have a high prevalence of malnutrition consequent to chronic diseases such as DM, depression, and dementia (15). Furthermore, malnutrition can increase the morbidity associated with these diseases and exacerbate the prognosis of existing diseases. In addition, deteriorating health in elderly women escalates medical bills (16). Despite this, scant research attention has been directed toward a comprehensive evaluation of the relationships of mobility with physical, psychological, or cognitive factors relative to nutrition status among elderly women with DM. Therefore, the present study aimed to investigate the factors influencing mobility relative to nutrition status in this population.

## Materials and Methods

### Study procedure and participants

Cross-sectional survey data were obtained from the Korean Elderly Adults Survey, conducted by the Korea Institute for Health and Social Affairs (KIHASA) from Jun to Sep 2014.

Written approval for the use of the survey data was obtained from KIHASA.

The original data were stratified by the seven metropolitan cities and nine provinces of South Korea. The 9 provinces were further divided into 18 classes by division into neighborhoods and towns/townships.

**Fig. 1:**
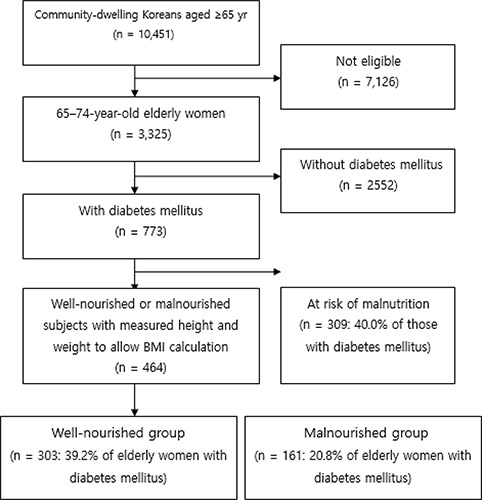
Subject flow

The Korean Elderly Adults Survey is conducted every 3 years. The scope of the 2014 survey was determined using responses from a panel of households surveyed in 2011. The procedure used to select the final study subjects is displayed in Fig. 1. A total minimum sample size of 109 (well-nourished group) and 103 (malnourished group) was required to ensure a medium effect size with α-value of 0.05 and power of 0.80 using *F*-tests (8 predictors for the well-nourished, 7 for the malnourished group). This effect size was derived using the G*Power sample size calculation engine 3.1.7 (17).

### Instruments/Difficulty with daily activities due to loss of visual acuity

This study investigated all diabetes-related causes of visual acuity loss, as well as others. Moreover, regarding vision aids (glasses, lenses, and reading glasses), study subjects were asked to give a score of 3 points if they felt very uncomfortable while performing daily activities, 2 points if they felt a little uncomfortable, and 1 point if they did not feel discomfort.

### Nutritional status

This study used the Korean version of the Nutritional Screening Initiative (NSI) checklist, translated and modified (18) to adapt to the Korean context, to examine the nutritional status of elderly people. The NSI checklist was developed in collaboration with 30 relevant associations, including the American Academy of Family Physicians, American Dietetic Association, and National Council on Aging in the United States. The NSI checklist includes 10 differently weighted yes/no items associated with the nutritional well-being of older people. The cumulative scores range from 0 to 21. In this study, patients with total scores of 0–2 belonged to the well-nourished group; those with scores ≥6 were classified into the malnourished group. The Cronbach’s α-values for this measure were 0.75 in a prior study (18) and 0.72 in the present study.

### Cognitive function

To evaluate the cognitive function, we used the Mini-Mental Status Examination from the Korean version of the CERED assessment packet (MMSE-KC) (19). Items in this examination address orientation to time and place (10 points), immediate recall (3 points), attention (5 points), delayed recall (3 points), language ability (6 points), configuration capability (1 point), and comprehension and judgment (2 points). Total scores can range from 0 to 30 points, with higher scores indicating higher levels of cognition. Summary scores were evaluated relative to norms for sex, educational level, and age. The MMSE-KC was standardized and found that the κ coefficient, which indicated agreement between the psychiatrist’s diagnosis and the MMSE-KC score, was 0.63 (19). In this study, the Cronbach’s α-value was 0.80.

### Depressive symptoms

Depressive symptoms were assessed using the Geriatric Depression Scale-Short Form (GDS-SF) developed (20) and modified (21). This scale was applied in Korea after its reliability and feasibility were confirmed. The GDS-SF comprises 15 items associated with critical thinking, feelings of unhappiness, decline in cognitive function, loss of energy, physical weakness, increasing hypochondria, social withdrawal, and loss of interest. All questions have dichotomous responses (“yes” = 1 point, “no” = 0 points), and total scores range from 0 to 15 points. A cut-off score of 5 points is used to indicate depression, and higher scores indicate more severe depressive symptoms. The Cronbach’s α-values were 0.89 in a prior study (21) and 0.91 in the present study.

### Muscle strength

To measure muscle strength, study subjects were asked to stand up and sit down in a chair 5 times with their hands clasped together in the forward direction. Scores were classified as complete performance (3 points), imperfect performance despite attempt (2 points), and inability to attempt (1 point). This measurement was reported to be useful and convenient for evaluating muscular strength and its uses in the basic activities of daily living in Korean elderly people (10).

### Mobility

The mobility scale (22) comprised 5 items: (1) reaching up over one’s head; (2) lifting or carrying an 8-kg bag of rice; (3) walking 400 meters; (4) climbing 10 stairs without stopping; and (5) stooping, crouching or kneeling. The items were scored from 0 to 3 depending on the degree of difficulty experienced while performing the task: 0 = unable to perform without help, 1 = significant difficulty, 2 = some difficulty, and 3 = no difficulty. The total score was divided by the number of questions, by 3 points, and then multiplied by 100 for a total possible score of 100. Higher scores indicated better function. The Cronbach’s α-values were 0.87 in the study that developed this scale and 0.87 in the present study.

### Statistical analyses

All data were analyzed using SPSS for Windows, ver. 18.0 (SPSS, Inc., Chicago, IL, USA). The threshold of significance was set at *P* <0.05. Weight was used to reduce selection bias and ensure the representativeness of the sample. A χ^2^-test was performed to verify the homogeneity of the participants’ sociodemographic factors. An independent-sample t-test was conducted to assess whether the main variables differed with respect to nutritional status. Correlations between these variables were calculated using Pearson’s correlation coefficient. Each variable that correlated significantly with mobility was regarded as an independent variable in a multiple linear regression analysis. A simultaneous data-entry method was employed.

## Results

### Sociodemographic characteristics in relation to nutritional status

The mean (± standard deviation; SD) age of the subjects was 69.6 ± 2.96 yr; 48.1% (n = 223) of the patients were 65–69 yr of age, and 51.9% (n = 241) were 70–74 yr of age. The majority (74.6%, n = 346) of the subjects had ≤6 yr of education, 42.9% (n = 199) were obese (BMI ≥25), and 93.1% (n = 432) exercised ≥3 d a week. There were no significant differences in sociodemographic characteristics between the well-nourished group and the malnourished group. Therefore, the groups were deemed homogeneous (Table 1).

**Table 1:** Sociodemographic characteristics relative to nutritional status (n = 464)

***Variable***	***Total (n =464) n (weighted %)***	***Well-nourished group (n =303) n (weighted %)***	***Malnourished group (n = 161) n (weighted %)***	**χ^2^*(*P*)***
Age (yr)				2.074 (0.172)
65–69	223 (48.1)	153 (50.5)	70 (43.5)	
70–74	241 (51.9)	150 (49.5)	91 (56.5)	
Mean ± SD	69.6 ± 2.96			
Educational level (yr)				9.815 (0.070)
6	346 (74.6)	212 (70.0)	134(83.2)	
7–12	103 (22.2)	79 (26.1)	24 (14.9)	
13	15 (3.2)	12 (4.0)	3 (1.9)	
Body mass index (kg/m^2^)				6.212 (0.102)
<18.5	14 (3.0)	8 (2.6)	6 (3.7)	
18.5–22.9	121 (26.1)	82 (27.1)	39 (24.2)	
23.0–24.9	130 (28.0)	94 (31.0)	36 (22.4)	
25.0	199 (42.9)	119 (39.3)	80 (49.7)	
Exercise frequency				3.858 (0.055)
<3 d a week	32 (6.9)	26 (8.6)	6 (3.7)	
3 d a week	432 (93.1)	277 (91.4)	155 (96.3)	

SD: standard deviation

### Mean differences of variables relative to nutritional status

The well-nourished and malnourished groups differed significantly regarding difficulty performing daily activities because of visual acuity loss (t= −4.817; *P*<0.001), muscle strength (t=6.382; *P*<0.001), depressive symptoms (t= −10.272; *P*<0.001), and mobility (t=9.601; *P*<0.001). There was no significant difference in cognitive function (t=1.852; *P*=0.066; Table 2).

**Table 2:** Mean differences of variables relative to nutritional status (n = 464)

***Variables***	***Well-nourished group (n = 303) NSL: 1.09 ± 0.77***	***Malnourished group (n = 161) NSL: 7.83 ± 1.99***	**t *(*P*)***
	Mean ± SD	Mean ± SD	
Difficulty of daily activities due to visual acuity loss	1.39 ± 0.54	1.68 ± 0.66	−4.817(<0.001)
Muscle strength	2.88 ± 0.35	2.57 ± 0.57	6.382 (<0.001)
Cognitive function	24.52 ± 3.90	23.04 ± 9.74	1.852 (0.066)
Depressive symptoms	4.74 ± 4.23	8.96 ± 4.20	−10.272 (<0.001)
Mobility	86.14 ± 16.71	66.29 ± 23.23	9.601 (<0.001)

NSL: nutritional status level, SD: standard deviation

### Correlations between mobility and other variables relative to nutritional status

Correlations of mobility with other variables relative to nutritional status are shown in Table 3. In the well-nourished group, mobility exhibited a significant negative correlation with difficulty performing daily activities because of visual acuity loss (r= −0.155; *P=*0.007), and depressive symptoms (r= −0.253; *P<*0.001). Furthermore, mobility was significantly positively correlated with muscle strength (r= 0.434; *P<*0.001) and cognitive function (r=0.139; *P=*0.015).

**Table 3:** Correlations between mobility and other variables relative to nutritional status (n=64)

***Variables***	***Mobility***	
	***Well-nourished group (n = 303)/r (P)***	***Malnourished group (n = 161)/r (P)***
Difficulty of daily activities due to visual acuity loss	−0.155 (0.007)	−0.011 (0.895)
Muscle strength	0.434 (<0.001)	0.563 (<0.001)
Cognitive function	0.139 (0.015)	0.110 (0.163)
Depressive symptoms	−0.253 (<0.001)	−0.272 (<0.001)
Nutritional status level	−0.046 (0.424)	−0.269 (0.001)

In the malnourished group, mobility was significantly negatively correlated with depressive symptoms (r= −0.272; *P<*0.001) and nutritional status (r= −0.269; *P=*0.001).

Mobility was significantly positively correlated with muscle strength (r=0.563; *P<*0.001).

### Factors influencing mobility relative to nutritional status

In the well-nourished group, BMI (*β*= −0.107; t= −2.104; *P*=0.036), difficulty in daily activities because of visual acuity loss (*β* = −0.110; t= −2.114; *P*=0.035), muscle strength (*β* = 0.385; t=7.334; *P*<0.001), and depressive symptoms (*β*= −0.233; t= −4.913; *P*<0.001) were significant predictors of mobility; the explanatory power of this model was 24.0% (*F*= 12.905; *P*<0.001) (Table 4).

**Table 4:** Factors influencing mobility relative to nutritional status (n = 464)

***Variable***	***Mobility***			
	Well-nourished group (n = 303)		Malnourished group (n = 161)	
	*β*	*t* (*P*)	*β*	*t* (*P*)
Constant		11.629 (<0.001)		8.620 (<0.001)
Age	−0.097	−1.901 (0.058)	−0.106	−1.705 (0.090)
Educational level	0.103	1.887 (0.060)	0.034	0.546 (0.586)
Body mass index	−0.107	−2.104 (0.036)	−0.005	−0.074 (0.941)
Exercise frequency	0.031	0.606 (0.545)	0.122	1.945 (0.054)
Difficulty of daily activities due to visual acuity loss	−0.110	−2.114 (0.035)		-
Muscle strength	0.385	7.334 (<0.001)	0.528	8.541 (<0.001)
Cognitive function	0.005	0.086 (0.932)		-
Depressive symptoms	−0.233	−4.913 (<0.001)	−0.174	−2.693 (0.008)
Nutritional status level		-	−0.217	−3.320 (0.001)
Adj. *R^2^*		0.240		0.405
*F* (*P*)		12.905 (<0.001)		16.589 (<0.001)

Adj.: adjusted

In the malnourished group, muscle strength (*β*= 0.528; t= 8.541; *P*<0.001), depressive symptoms (*β* = −0.174; t= −2.693; *P*=0.008), and nutritional status (*β* = −0.217; t= −3.320; *P*=0.001) were significant predictors of mobility; the explanatory power of this model was 40.5% (*F*=16.589; *P*<0.001).

## Discussion

The current study aimed to gain a better understanding of the factors influencing mobility relative to nutritional status in older women with DM. For older women, mobility is critical in maintaining an independent daily life and social interactions; for this and other reasons, it is an important public health issue. Unfortunately, little is known about the relationships among physical, psychological, and cognitive factors relative to nutritional status in older women with DM.

The findings in this study indicate that the malnourished group experienced more difficulty because of visual acuity loss while performing daily activities, compared to the well-nourished group. Sensory dysfunction, including vision loss, can cause disabilities in mobility and cognitive function and may disrupt a patient’s ability to lead an independent life. Sensory dysfunction eventually degrades a person’s quality of life (23). Per the National Health and Nutrition Examination Surveys conducted in the United States (24), people with DM are more likely to develop uncorrectable visual impairments, compared to those without diabetes, even after controlling for selected other factors. Moreover, people with DM receive significantly lower scores on the Mini-Nutritional Assessment, compared to those without DM (25). Elderly people who have trouble when performing daily activities because of visual acuity loss can have trouble cooking, grocery shopping, and setting the table. This situation can cause a vicious circle, leading to malnourishment (26). Patients with DM are also at risk of developing diabetic retinopathy, leading to accelerated vision loss. Therefore, early diagnosis should be used to ensure that these patients receive appropriate intervention.

Regarding the measurement of muscular strength in elderly women with DM, the well-nourished group had better muscular strength than the malnourished group, perhaps because well-nourished elderly people generally have better muscle function than malnourished elderly people. Therefore, our results corroborate those of a previous study in which handgrip strength could independently predict the nutritional status of individuals (27). The loss of fast twitch fibers, protein glycation, and insulin resistance may all play important roles in the loss of muscle strength, as well as the development of sarcopenia (6). Protein intake is integral to muscle health and decreases in vitamin B_12_ and folic acid intake can impair muscle function, as these nutrients act on homocysteine (6). Therefore, adequate nutrition is important to the preservation of muscle mass and strength during aging. Accordingly, nutritional management is essential for elderly women.

A mean measured GDS score of 2.7 was observed among well-nourished Japanese senior citizens, whereas the corresponding score of malnourished subjects was 6.2 (28). Thus, the results of the previous study and our study were similar. However, in the present study, both groups received average depression scores that were 2 points higher (well-nourished group: 4.74; malnourished group: 8.96) than those of subjects in the prior study. A community-based study of Spanish elderly subjects demonstrated that DM was associated with an increased risk of incident (1.5 times) and prevalent (1.4 times) depression (29). Interestingly, the presence of comorbidities increased the risk of incident depression (30). Therefore, DM may play a role in the development of depression in the elderly.

Regarding elderly women with DM, the well-nourished group had a higher level of mobility than the malnourished group. For example, among 6588 community-dwelling individuals aged ≥60 yr, 32% of women were reported an inability to walk a quarter of a mile or climb stairs; the corresponding rate for women without diabetes was 14% (31). The outcome of the present study was similar to that found elderly people with disease-related malnutrition due to vitamin D deficiency may develop osteoporosis or experience decreased mobility (4). Therefore, when an elderly person falls, they are more likely to develop a disability.

We identified muscle strength and depressive symptoms as major factors that significantly influenced mobility in both groups. In a study cohort of 3075 well-functioning black and white men and women aged 70–79 yr, muscle attenuation and muscle strength were found to independently predict limited mobility (32). More specifically, a prospective cohort study of 4757 people from 4 European nations was reported “only depression” and “depression and anxiety” affect mobility (33); in other words, physical health was influenced by depression. Moreover, depressive symptoms affected life-space mobility in a study of community-dwelling older women (34). Therefore, the previous study corroborated our findings. The association between mobility and depressive symptoms is likely to be bidirectional in that each increases the probability of the other, potentially leading to a vicious circle. In the present study, only the nutritional status influenced elderly women with DM in the malnourished group. A previous study using the Mini Nutritional Assessment tool found that malnourished individuals performed more poorly than other groups in a short physical performance battery test (35), thus supporting the results of the current study. Endocrine disorders such as DM can increase the risk of malnutrition in the elderly (36). Particularly, persistent symptoms of hyperglycemia can cause serious damage to body structures (37). Elderly women with DM in the malnourished group will need to manage their health through aerobic exercise, blood glucose control, and systematic diet management.

This study had both strengths and limitations. It was based on a cross-sectional survey, and any causal inferences cannot be determined from the identified associations. Nevertheless, this was the first representative study to demonstrate the effects of physical function on the nutritional statuses of elderly women with DM.

## Conclusion

Therefore, clinicians should consider these factors when planning a program to improve mobility in such individuals. Moreover, it is difficult to improve an elderly person’s nutritional status by relying on their willpower. Accordingly, public health nurses must support improved nutrition among the elderly at a community level. Community nurses responsible for elderly health management should possess or develop professional capabilities that would facilitate their awareness of all factors relevant to malnutrition in elderly people and ability to assess and diagnose malnutrition in its early stages. The associations of life-space mobility with other variables should be studied further in longitudinal studies; in particular, we should examine temporality and potential causality within this system.

## Ethical considerations

The author completely observed and conformed to all ethical guidelines regarding plagiarism, informed consent, misconduct, data fabrication and/or falsification, double publication and/or submission, and redundancy.
